# Machine Learning-Based Prediction of 48-Hour Extubation Success in Mechanically Ventilated Children: A Single-Center Retrospective Cohort Study

**DOI:** 10.3390/children13091166

**Published:** 2026-08-29

**Authors:** Ferhat Sarı, Aynur Aliyeva

**Affiliations:** 1 Department of Pediatrics, Division of Pediatric Intensive Care, Faculty of Medicine, Istanbul Aydın University, Istanbul 34295, Turkey; ferhatsari_dr@hotmail.com; 2 Division of Otolaryngology, Department of Surgery, Denver Health Medical Center, Denver, CO 80204, USA; 3 Neuroscience Doctoral Program, Yeditepe University, Istanbul 34755, Turkey

**Keywords:** pediatric extubation, mechanical ventilation, machine learning, extubation success, pediatric intensive care unit

## Abstract

**Highlights:**

**What are the main findings?**

- In this retrospective cohort of 341 mechanically ventilated children, 298 (87.4%) achieved 48 h extubation success, while 43 (12.6%) experienced extubation failure.
- Random Forest showed the numerically highest internally cross-validated discrimination, with an AUC of 0.983 (95% CI, 0.971–0.993), while model-based analyses highlighted respiratory, metabolic, inflammatory, and neurological variables associated with extubation outcome.

**What are the implications of the main findings?**

- Integrating routinely available multidimensional pre-extubation data may improve risk stratification beyond reliance on any single conventional weaning parameter.
- These findings should currently be regarded as internal proof-of-concept evidence and require prospective multicenter external validation before routine clinical implementation.

**Abstract:**

**Background:** Accurate assessment of extubation readiness in mechanically ventilated children remains difficult because successful sustained breathing depends on the interaction of respiratory, metabolic, inflammatory, neurological, and cardiovascular factors. This study developed and internally validated machine-learning models for predicting 48 h extubation success using routinely available pre-extubation data. **Methods:** Of 1097 total PICU admissions, 341 mechanically ventilated children treated between 2021 and 2026 constituted the final analytic cohort. The primary outcome was survival without reintubation during the first 48 h after planned extubation. Demographic, clinical, laboratory, blood gas, illness severity, and ventilator variables were evaluated. Logistic Regression, Random Forest, Gradient Boosting, and Support Vector Machine models were assessed using stratified 5-fold cross-validation. Unsupervised k-means clustering was performed to identify physiological phenotypes. **Results:** Extubation was successful in 298 children (87.4%) and failed in 43 (12.6%). Failure was associated with higher oxygenation index, lactate, procalcitonin, C-reactive protein, PaCO_2_, pSOFA, PEEP, and rapid shallow breathing index, together with lower arterial pH, bicarbonate, ionized calcium, hemoglobin, albumin, sodium, and Glasgow Coma Scale scores. Random Forest yielded the numerically highest discrimination, with an AUC of 0.983 (95% CI, 0.971–0.993), a sensitivity of 0.980, a specificity of 0.814, an accuracy of 0.959, and a Brier score of 0.032. Arterial pH, the oxygenation index, bicarbonate, ionized calcium, procalcitonin, lactate, and PaCO_2_ showed the highest Random Forest Gini importance scores. Clustering identified a low-severity phenotype (*n* = 302; 97% success; 0% mortality) and a high-severity phenotype (n = 39; 10% success; 100% mortality). **Conclusions:** Multidimensional machine-learning models predicted 48 h pediatric extubation success with strong internal discrimination. Prospective multicenter external validation is required before clinical implementation.

## 1. Introduction

Invasive mechanical ventilation is a lifesaving intervention for children with acute respiratory failure and other critical illnesses; however, determining when ventilatory support can be safely discontinued remains a complex component of pediatric intensive care. Premature extubation may result in respiratory deterioration and reintubation, whereas unnecessary continuation of invasive ventilation increases exposure to ventilator-associated complications, airway injury, sedative medications, respiratory muscle dysfunction, and prolonged intensive care treatment [1,2].

Mechanical ventilation liberation includes readiness assessment, gradual support reduction, spontaneous breathing testing, and extubation. However, passing a spontaneous breathing trial does not guarantee success; success also depends on airway patency, neurological function, cough and secretion clearance, respiratory muscle reserve, gas exchange, and cardiovascular stability [1,2].

Prediction of extubation outcome is particularly challenging in pediatric intensive care because children constitute a physiologically heterogeneous population ranging from infants to adolescents. Age-dependent differences in airway dimensions, respiratory rates, lung and chest-wall mechanics, disease characteristics, nutritional status, and developmental physiology limit the direct application of adult-derived thresholds and complicate the establishment of pediatric extubation criteria [1,2,3,4].

Clinical extubation decisions therefore require integrating multiple domains, including the primary diagnosis, comorbidities, duration of mechanical ventilation, sedative exposure, neurological status, organ support, laboratory findings, arterial blood gases, ventilator settings, respiratory mechanics, and performance of spontaneous breathing trials. Conventional parameters such as respiratory rate, tidal volume, inspiratory force, oxygenation indices, and the rapid shallow breathing index may provide useful information, but their predictive performance in children is variable, and no single parameter is sufficiently reliable to independently determine extubation readiness [4,5,6,7].

Extubation failure is typically defined as reintubation within 24–48 h after planned extubation. It may result from unresolved pulmonary disease, respiratory muscle weakness, upper airway obstruction, neurological impairment, inadequate secretion clearance, cardiovascular instability, or any combination thereof [2,4,5]. Because extubation outcome reflects interacting physiological and clinical factors, multivariable models may outperform isolated thresholds. Logistic regression offers interpretability, whereas machine-learning methods can capture nonlinear relationships and complex interactions among demographic, clinical, laboratory, severity, and ventilator variables [6,8,9].

Greater algorithmic complexity does not necessarily improve accuracy, reliability, or clinical utility. Pediatric intensive care AI studies remain heterogeneous in design, populations, outcome definitions, validation, and reporting, and few models have undergone prospective evaluation or demonstrated patient-centered benefit [9,10,11]. A clinically credible model should use only pre-extubation data and exclude post-extubation outcomes. Evaluation should assess discrimination, precision–recall performance, calibration, internal validation, uncertainty, and comparison with prespecified benchmarks rather than accuracy alone [12,13,14,15].

This study aims to develop and internally validate machine-learning models to estimate the probability of 48 h extubation success using routinely collected pre-extubation demographic, clinical, laboratory, illness severity, and ventilator data. Secondary objectives are to compare the predictive performance of different algorithms, identify the variables that contribute most strongly to model predictions, and establish a reproducible foundation for subsequent external and prospective validation prior to any clinical implementation.

## 2. Materials and Methods

### 2.1. Study Design and Setting

**Figure 1:**
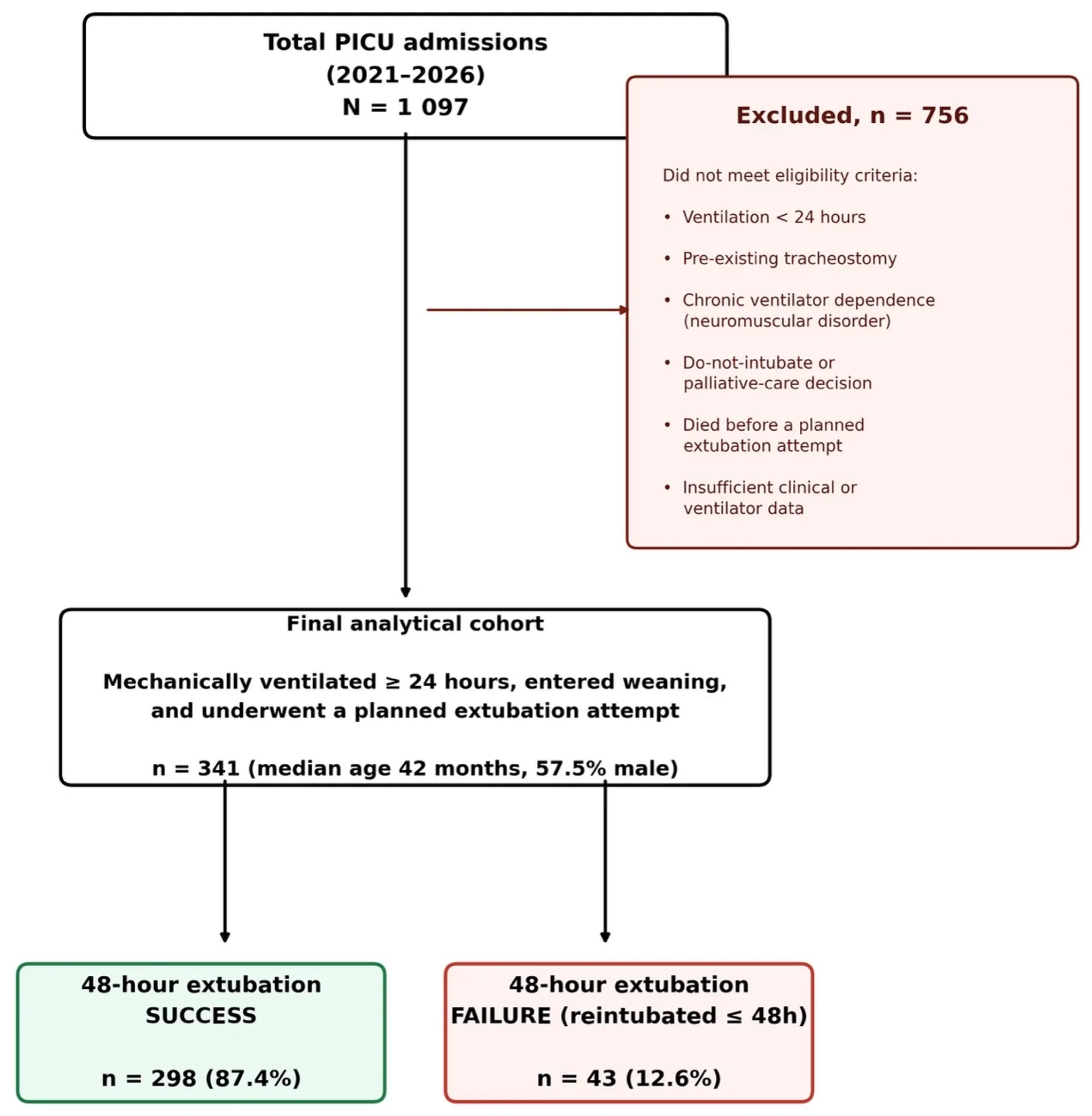
Cohort assembly and patient flow diagram.

This single-center, retrospective, observational cohort study included eligible PICU admissions from 2021 through 2026 and was conducted under retrospective archive-based ethical approval granted on 25 March 2026. During the study period, 1097 patients were admitted to the PICU, of whom 341 received invasive mechanical ventilation and constituted the source population for eligibility assessment. The study was designed to develop and internally validate machine-learning models for predicting extubation success using routinely collected clinical data. The approved protocol classified the investigation as a retrospective, archive-based, non-interventional study (Figure 1). Ethical approval was obtained from the Istanbul Aydın University Non-Interventional Clinical Research Ethics Committee (Decision No. 113/2026; 25 March 2026)

### 2.2. Study Population

**Figure 2:**
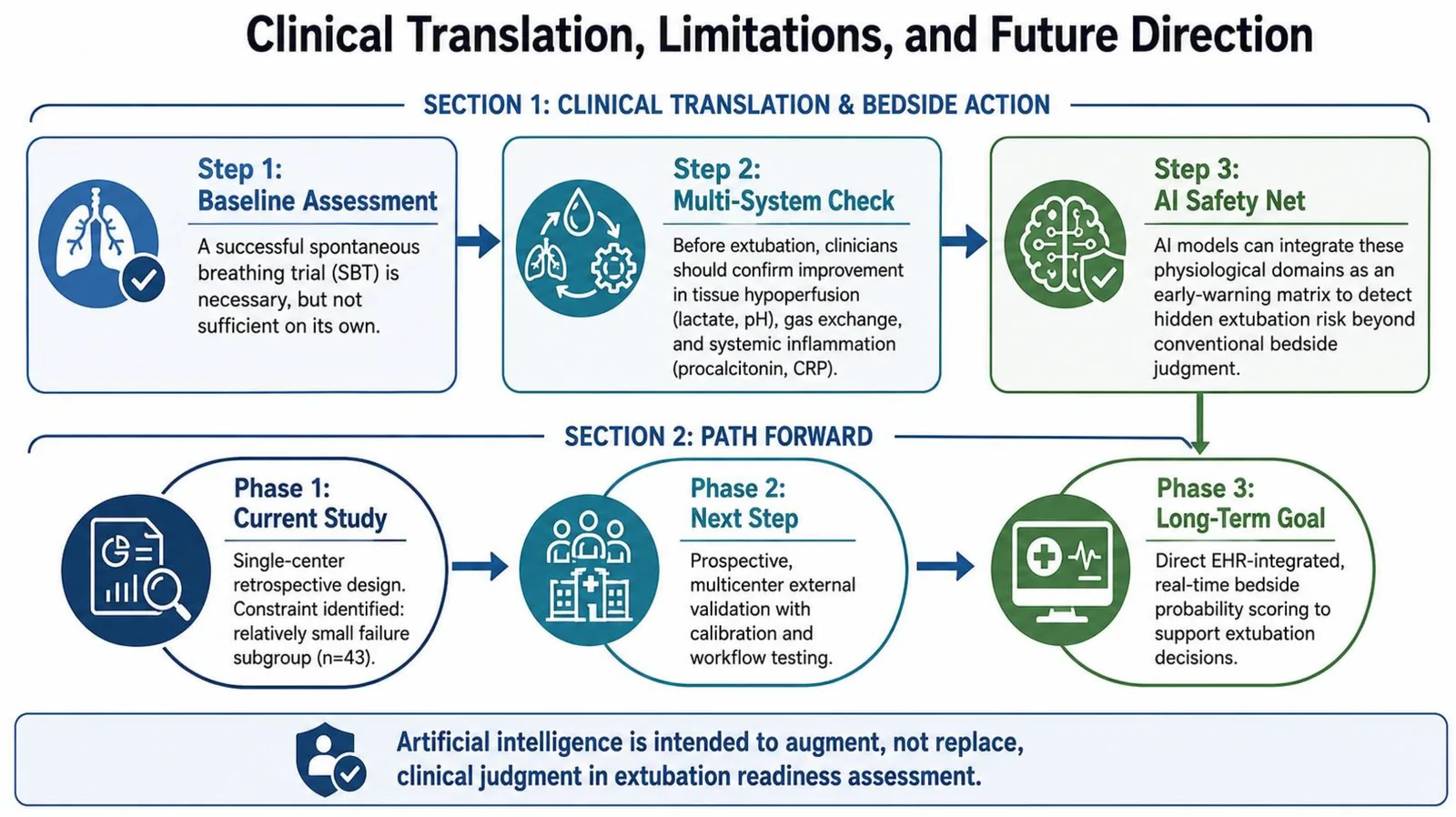
Clinical translation, limitations, and future direction.

**Figure 3:**
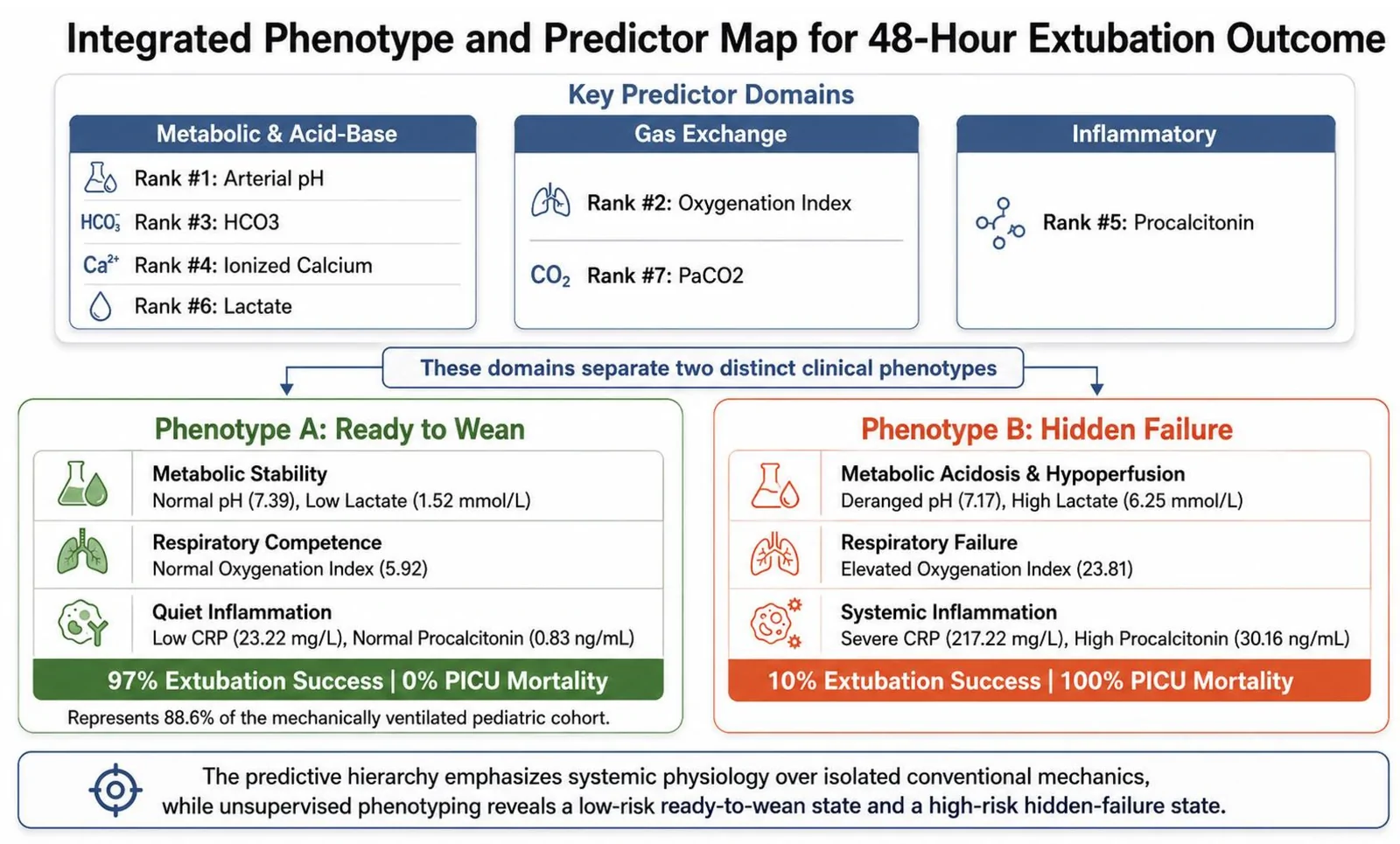
Integrated phenotype and predictor map for 48-h extubation outcome.

Patients aged 0–18 years were eligible when they had received invasive mechanical ventilation for at least 24 h, entered a ventilator-weaning process, and subsequently underwent a planned extubation attempt. Patients were excluded if they received mechanical ventilation for less than 24 h, had a pre-existing tracheostomy, had chronic ventilator dependence caused by a neuromuscular disorder, were managed under do-not-intubate or palliative-care decisions, or had insufficient clinical or ventilator data to determine the study outcome. Only patients who underwent a documented planned extubation attempt were included in the analytic cohort; palliative, compassionate, or terminal extubations and patients managed under do-not-intubate decisions were excluded. Patients who died before undergoing a planned extubation attempt were therefore not eligible for inclusion. Unplanned, accidental, or self-extubations were excluded from the analytic cohort (Figure 1). An overview of the clinical translation pathway and integrated predictor-phenotype summary is provided in Figure 2 and Figure 3. 

All included cases represented documented planned extubation attempts; palliative, compassionate, and terminal extubations, as well as patients managed under do-not-intubate decisions, were excluded. The retrospective dataset recorded SBT type, duration, and number of trials but did not include a separate binary variable indicating formal SBT pass/failure status or the clinician-specific indication for proceeding with extubation.

### 2.3. Data Collection and Candidate Predictors

Data were extracted from the hospital electronic health record system and the PICU database using a standardized data collection form. *Candidate predictors* included age, sex, body weight, nutritional z score, primary diagnostic category, chronic lung disease, duration of mechanical ventilation up to the prediction point, sedation exposure up to the prediction point, neuromuscular-blocking agent use, vasoactive treatment, Vasoactive–Inotropic Score, sepsis, acute kidney injury, continuous renal replacement therapy, heart rate, respiratory rate, Glasgow Coma Scale score, and sedation score.

*Laboratory and arterial blood-gas* variables included hemoglobin, C-reactive protein, procalcitonin, albumin, lactate, pH, PaCO_2_, bicarbonate, sodium, potassium, calcium, magnesium, phosphorus, PaO_2_, FiO_2_, PaO_2_/FiO_2_ ratio, and arterial oxygen saturation. Respiratory and ventilator variables included positive end-expiratory pressure, peak inspiratory pressure, maximum peak inspiratory pressure, tidal volume indexed to body weight, the rapid shallow breathing index, and the oxygenation index.

*Weaning-related variables* comprised weaning method, spontaneous breathing trial type, trial duration, and number of spontaneous breathing trials. Total weaning duration was excluded from model development because its temporal definition relative to the prediction point could not be established consistently. Illness severity was assessed using the Pediatric Risk of Mortality IV (PRISM IV) score, PRISM IV-predicted mortality percentage, and the pediatric Sequential Organ Failure Assessment (SOFA) score. Only variables available before planned extubation were considered candidate predictors.

*The prediction time* was defined as the clinical assessment immediately preceding planned extubation. For repeatedly measured laboratory, blood-gas, neurological, and ventilator variables, the most recent documented value available before the extubation decision was used. When multiple measurements were available on the same day, the value recorded closest to the extubation decision was selected. Exact measurement-to-extubation intervals were not consistently available in the retrospective dataset.

Only information available up to the defined pre-extubation prediction point was eligible for model development. Duration variables included in model development, including mechanical ventilation and sedation duration, were restricted to information available at the prediction point, whereas post-extubation variables, including PICU and hospital length of stay, reintubation timing, post-extubation respiratory support, tracheostomy, and mortality, were treated exclusively as outcomes and were not entered into the predictive models.

### 2.4. Outcomes

*The primary outcome* was 48 h extubation success, defined as survival without reintubation during the first 48 h after planned extubation. *Secondary outcomes* included reintubation, time to reintubation, noninvasive ventilation after extubation, post-extubation stridor, tracheostomy during the index PICU admission, duration of mechanical ventilation, PICU length of stay, hospital length of stay, and PICU mortality. The primary outcome was assessed specifically during the first 48 h after planned extubation. Reintubation occurring after this 48-h window was recorded as a secondary outcome and did not constitute failure of the prespecified 48 h primary endpoint. Deaths within 48 h without reintubation were classified as primary-endpoint failures, whereas deaths occurring after 48 h did not alter the 48 h extubation outcome classification.

### 2.5. Statistical and Machine-Learning Analysis

Continuous variables were summarized as mean ± SD or median [IQR] and categorical variables as n (%). Group comparisons used Student’s *t*-test or the Mann–Whitney U test and chi-square or Fisher’s exact test as appropriate. Four supervised classifiers, Logistic Regression, Random Forest, Gradient Boosting, and Support Vector Machine, were evaluated using stratified 5-fold cross-validation. Uncertainty around model performance was quantified using 95% bootstrap percentile confidence intervals from 3000 stratified bootstrap resamples of pooled out-of-fold predictions, while preserving the observed success/failure class distribution. Confidence intervals were calculated for the ROC-AUC, accuracy, sensitivity, specificity, positive predictive value, the F1 score, and the Brier score using a classification threshold of 0.50, with extubation success defined as the positive class. Precision–recall performance was additionally evaluated for extubation failure as the positive class using average precision (PR-AUC), with 95% bootstrap confidence intervals and comparison with the no-skill baseline defined by the observed failure prevalence. Analyses were conducted using Python (version 3.12.7), SPSS (version 29.0.2.0), and R (version 4.5.1), with two-sided *p* < 0.05 considered significant.

Nominal categorical predictors, including primary diagnosis, weaning method, and SBT type, were one-hot encoded. All preprocessing was performed within each training fold. Continuous predictors for Logistic Regression and SVM were standardized using training-fold parameters, and SVM probability estimates were obtained using sigmoid probability calibration fitted within the training data. Stratified five-fold cross-validation used a fixed random seed (random_state = 42). The same prespecified pre-extubation predictor set was used across all supervised models, with post-extubation variables excluded. Fixed model specifications were L2-regularized Logistic Regression, Random Forest (500 trees; maximum depth 5), Gradient Boosting (200 estimators; maximum depth 2), and RBF-kernel SVM, with no separate hyperparameter search. The final 341-patient analytic cohort contained complete data for all model predictors, and no additional complete-case exclusions or imputation were required. Complete availability of every candidate predictor was not an inclusion criterion, and no otherwise eligible patient was excluded solely because of missing model-predictor values.

Performance uncertainty was estimated using 95% bootstrap percentile confidence intervals from 3000 stratified bootstrap resamples that preserve the observed class distribution, with a 0.50 classification threshold and extubation success as the positive class. These bootstrap intervals quantify uncertainty in the pooled out-of-fold predictions but do not fully capture variability arising from model refitting or from the specific allocation of cross-validation folds. Calibration of the Random Forest model was assessed on pooled out-of-fold predictions using a logistic calibration model, yielding a calibration slope of 1.074 and calibration-in-the-large of −0.010 (slope = 1/intercept = 0 indicating perfect calibration)

K-means clustering was applied to 12 standardized pre-extubation variables: the oxygenation index, lactate, procalcitonin, CRP, pH, PaCO_2_, bicarbonate, pSOFA, PRISM-IV score, GCS, the RSBI, and PEEP; none of these variables contained missing data. The two-cluster solution was selected based on silhouette analysis (k = 2–5); PCA was used for visualization, and cluster stability was assessed using bootstrap resampling (500 iterations; mean Adjusted Rand Index 1.000, 95% CI 1.000–1.000) and comparison with two alternative clustering algorithms, hierarchical agglomerative clustering (ARI = 1.000) and a Gaussian mixture model (ARI = 0.982), applied to the same 12 standardized variables. In addition to Gini importance, permutation importance was calculated on held-out cross-validation folds (30 repeats per fold) to obtain a less biased estimate of individual predictor contribution, since Gini importance favors continuous variables with more potential split points.

Calibration of the Random Forest was assessed from pooled out-of-fold predicted probabilities using calibration-in-the-large, calibration slope, a calibration plot, and the Brier score. The Brier score was additionally compared with a prevalence-only model assigning the observed event probability to all patients.

## 3. Results

This retrospective cohort comprised 341 mechanically ventilated children admitted to a pediatric intensive care unit (PICU) between 2021 and 2026. An overview of the study framework, clinical translation pathway, and integrated predictor-phenotype summary is provided in Figure 1, Figure 2 and Figure 3. The median age was 42 months (IQR 12–119), and the median weight was 13.4 kg (IQR 7.9–25.0); 57.5% of patients were male. The most frequent primary diagnostic categories were respiratory disease (29.3%), neurological disease (26.1%), and no major comorbidity (24.6%), followed by infection/sepsis (19.1%) and cardiac disease (0.9%); 12.0% had chronic lung disease. No patient had a pre-existing tracheostomy at baseline. Weaning was achieved predominantly through gradual pressure reduction in invasive mode (49.3%) or CPAP (44.6%), with T-piece trials used less frequently (6.2%). Overall, 298 of 341 patients (87.4%) achieved 48 h extubation success, whereas 43 (12.6%) experienced extubation failure. During the overall follow-up period, 64 patients (18.8%) underwent reintubation; 43 were reintubated within 48 h, and 21 were reintubated after the 48 h primary-outcome window. Post-extubation stridor occurred in 13.5% (46/341), new tracheostomy was performed in 11.4% (39/341), and PICU mortality was 11.4% (39/341) (Table 1). Among the 39 patients who died during the PICU stay, 35 had undergone reintubation within 48 h, two were reintubated after 48 h, and two died without recorded reintubation. The retrospective dataset did not contain sufficiently granular time-to-death information to determine whether the two deaths without reintubation occurred within or after the 48 h primary-outcome window.

*Bivariate comparison* (Mann–Whitney U for continuous variables, chi-square/Fisher exact for categorical variables) showed that extubation outcome was primarily associated with markers of respiratory, metabolic, inflammatory, neurological, and overall illness severity, including the oxygenation index, lactate, procalcitonin, CRP, arterial pH, PaCO_2_, bicarbonate, ionized calcium, GCS, pSOFA, PEEP, the RSBI, and PRISM-IV (all *p* < 0.001). Primary diagnosis (*p* < 0.001) and chronic lung disease (*p* = 0.030) were the only significant categorical factors, whereas demographic characteristics, ventilation duration, weaning method, and most treatment-related variables were not associated with outcome (Table 2).

**Figure 4:**
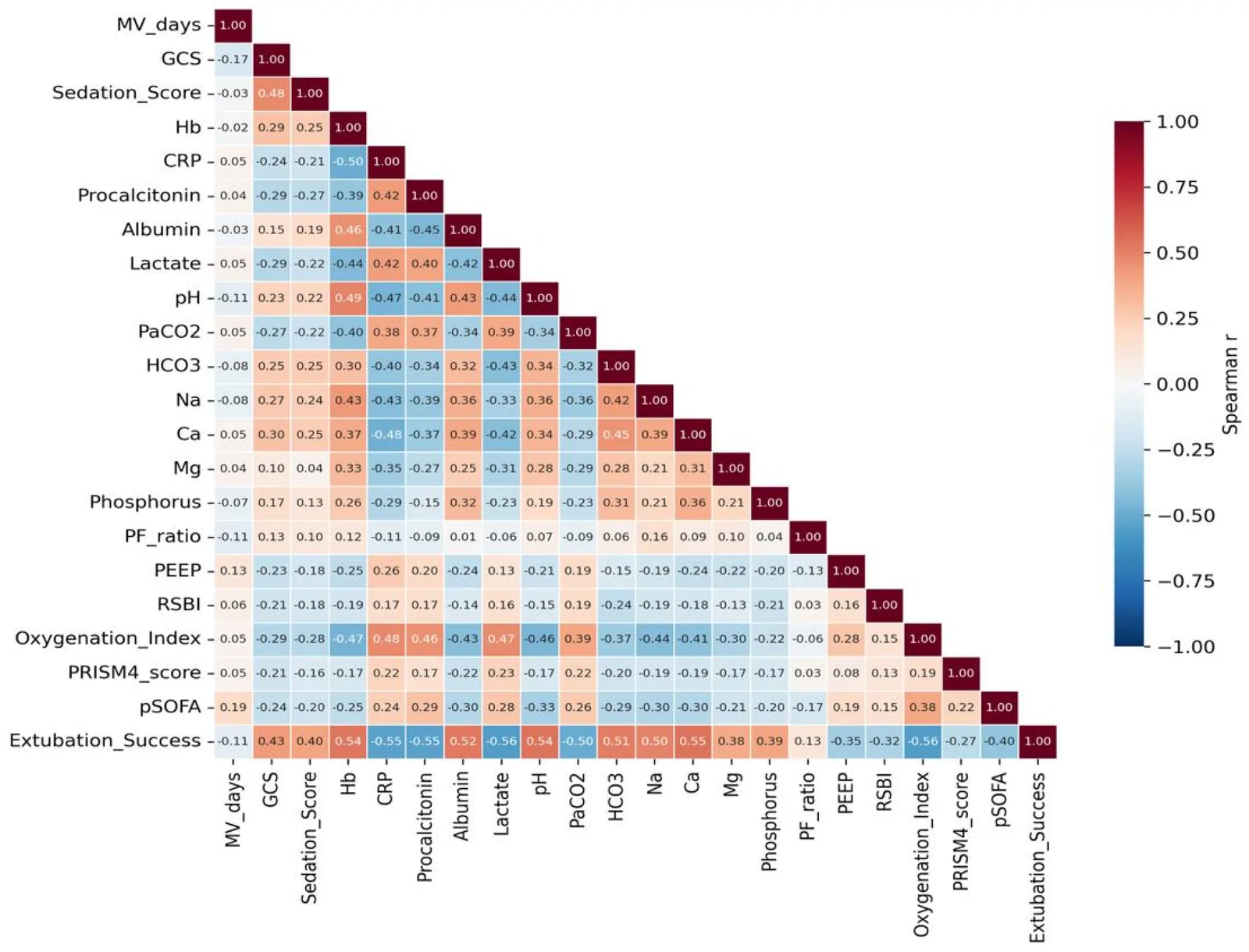
Spearman correlation matrix of key clinical, laboratory, and ventilator parameters with 48 h extubation success.

*Spearman correlation analysis* (Figure 4) confirmed this pattern: extubation success correlated negatively with oxygenation index (r = −0.56), lactate (r = −0.56), procalcitonin (r = −0.55), C-reactive protein (r = −0.55), PaCO_2_ (r = −0.50), pSOFA (r = −0.40), PEEP (r = −0.35), RSBI (r = −0.32), and PRISM-IV score (r = −0.27) and positively with arterial pH (r = 0.54), ionized calcium (r = 0.55), hemoglobin (r = 0.54), albumin (r = 0.52), bicarbonate (r = 0.51), sodium (r = 0.50), the Glasgow Coma Scale (r = 0.43), and sedation score (r = 0.40). No meaningful correlation was observed for age, weight, or malnutrition z-score, indicating that weaning outcome in this cohort was driven predominantly by acute physiological derangement rather than baseline anthropometric characteristics.

**Figure 5:**
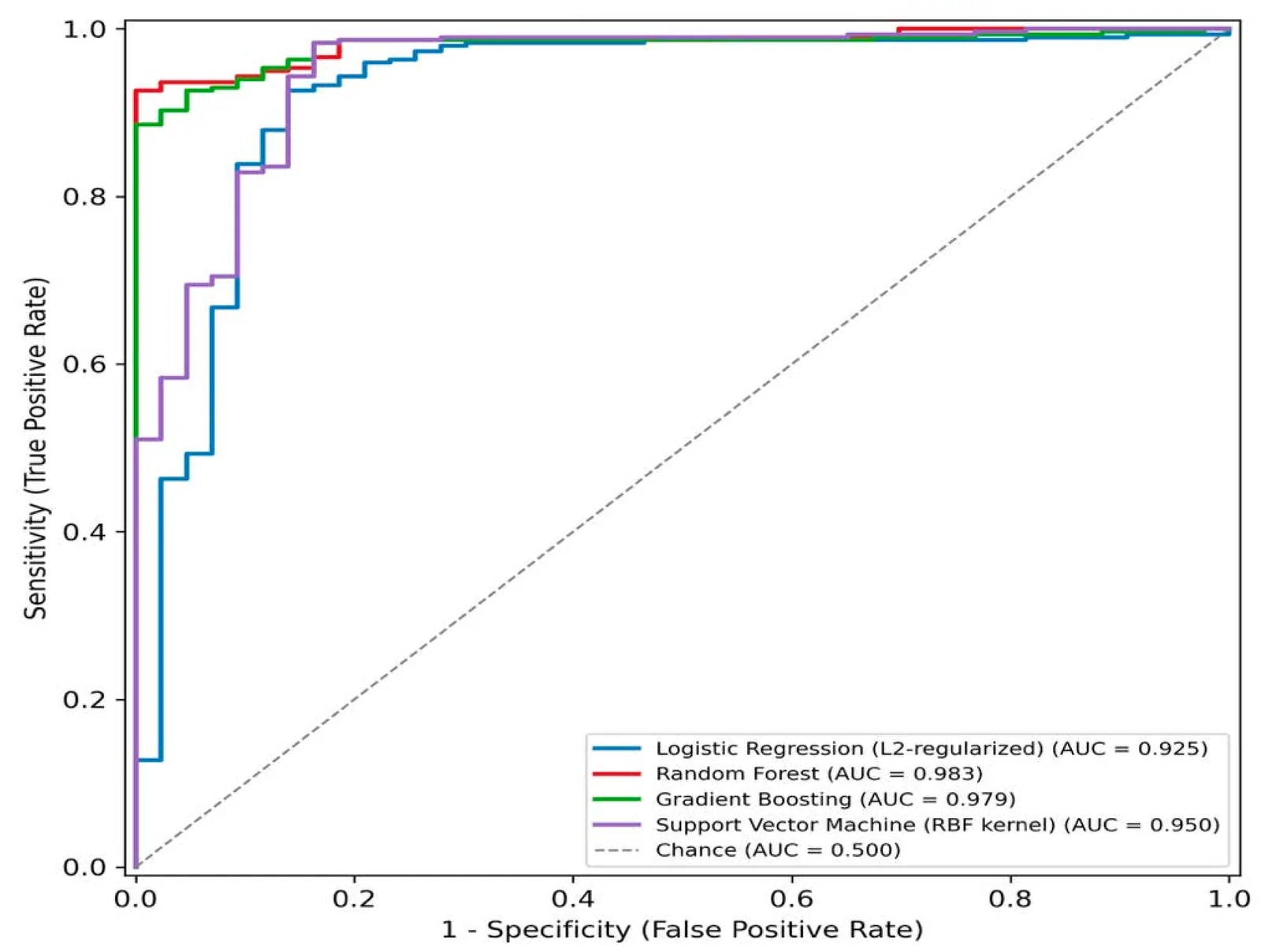
Five-fold cross-validated ROC curves comparing Logistic Regression, Random Forest, Gradient Boosting, and Support Vector Machine models for prediction of 48 h extubation success.

*Four named supervised machine-learning* classifiers were trained to predict 48 h extubation success using clinical, laboratory, and ventilator parameters, with performance assessed by stratified 5-fold cross-validation (Table 3, Figure 5): (1) L2-regularized Logistic Regression, (2) Random Forest (500 trees, max depth 5), (3) Gradient Boosting (200 estimators, max depth 2), and (4) Support Vector Machine with a radial basis function (RBF) kernel. All four models demonstrated strong discrimination. Among the evaluated models, Random Forest yielded the numerically highest cross-validated AUC of 0.983 (95% CI, 0.971–0.993), followed by Gradient Boosting at 0.975 (95% CI, 0.955–0.990), Support Vector Machine at 0.953 (95% CI, 0.914–0.983), and Logistic Regression at 0.924 (95% CI, 0.866–0.971). Random Forest achieved an accuracy of 0.959 (95% CI, 0.938–0.977), a sensitivity of 0.980 (95% CI, 0.963–0.993), a specificity of 0.814 (95% CI, 0.698–0.930), and a Brier score of 0.032 (95% CI, 0.020–0.045). Fold-level ROC-AUC values for all four supervised models are provided in Table 3. Across the five validation folds, AUCs ranged from 0.844 to 1.000 for Logistic Regression, 0.966 to 1.000 for Random Forest, 0.966 to 1.000 for Gradient Boosting, and 0.923 to 1.000 for Support Vector Machine. The reported pooled AUCs were calculated from pooled out-of-fold predictions and should be interpreted as exploratory internal performance estimates. The pooled AUC of 0.983 should therefore be regarded as an exploratory internal performance estimate rather than a definitive estimate of generalizable performance. Support Vector Machine yielded the numerically highest accuracy, 0.965 (95% CI, 0.944–0.982), and sensitivity, 0.987 (95% CI, 0.973–0.997) (Table 3).

**Figure 6:**
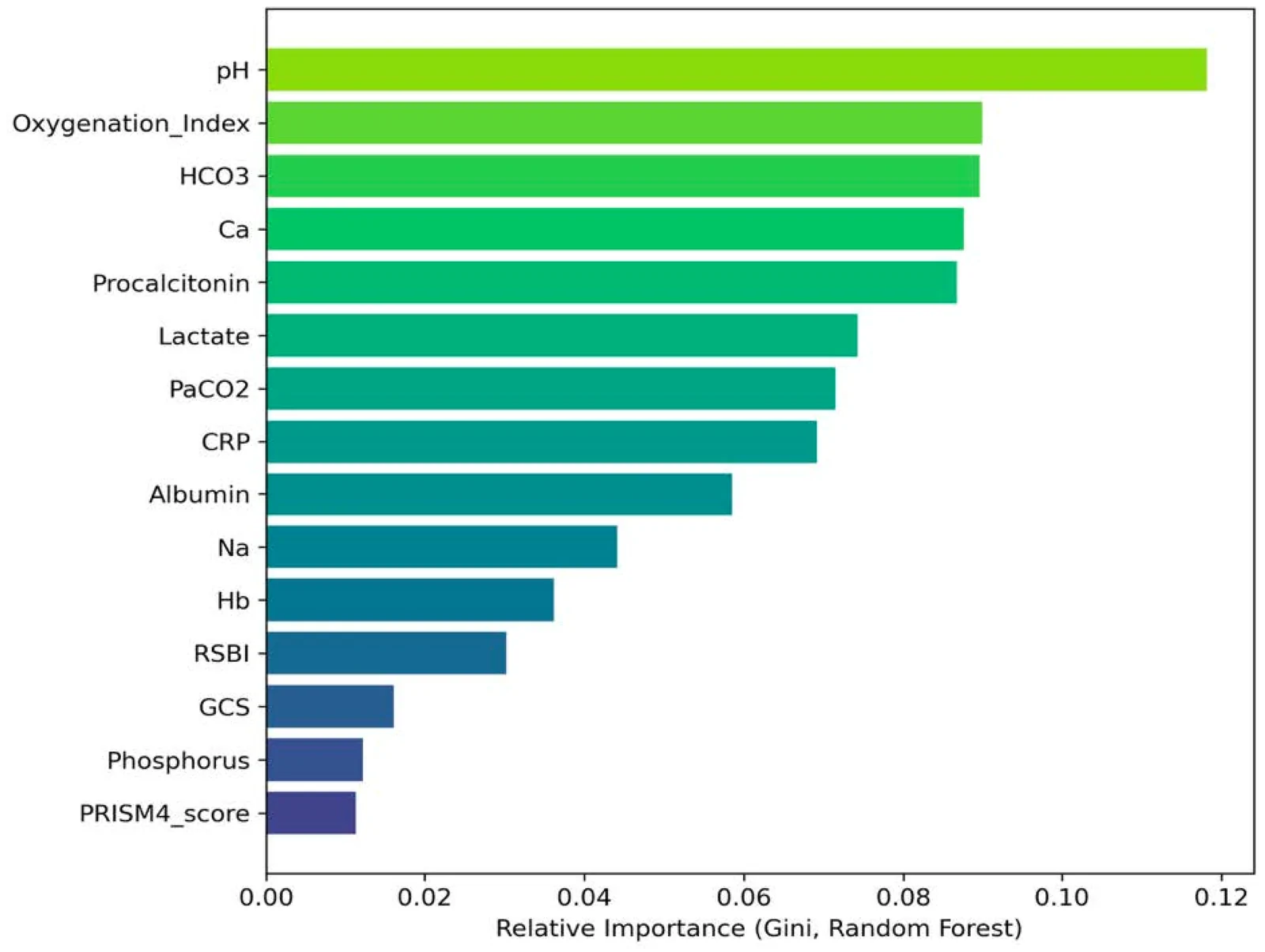
Top 15 Random Forest Gini feature-importance scores for 48-h extubation success.

*Random Forest* Gini importance assigned the highest importance scores to arterial pH, the oxygenation index, bicarbonate, ionized calcium, procalcitonin, lactate, and PaCO_2_, followed by C-reactive protein, albumin, sodium, hemoglobin, and the RSBI. These rankings represent model-based feature importance and should not be interpreted as independent or causal effects. (Figure 6, Table 4). Given the limited number of extubation-failure events and the substantial correlation among candidate predictors, no independent-effect interpretation was pursued beyond the supervised predictive analyses and descriptive assessments of feature importance. (Table 4).

Permutation importance calculated on held-out folds produced a materially different ranking (Table 5): the RSBI showed the most robust individual contribution (mean importance 0.0040, 95% CI 0.0009–0.0072), followed by arterial pH (0.0026, 95% CI −0.0026 to 0.0078) and lactate (0.0016, 95% CI 0.0000–0.0031). Arterial pH and the oxygenation index, despite ranking first and second by Gini importance, showed permutation-importance confidence intervals that crossed zero, indicating that their individual predictive contributions were not consistently distinguishable from zero in held-out observations (Table 5).

**Figure 7:**
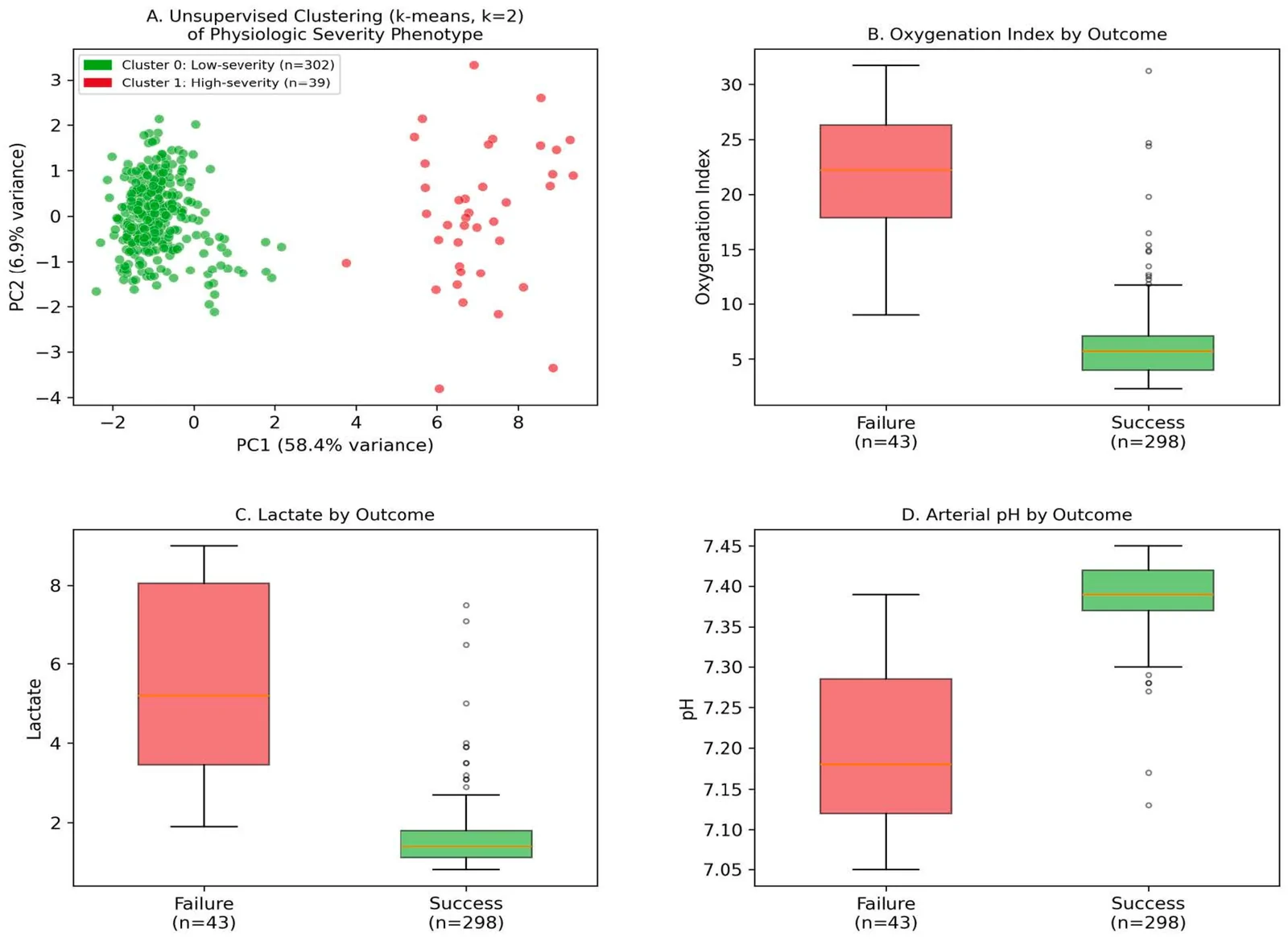
Physiologic severity phenotypes and extubation outcome profiles.

**Figure 8:**
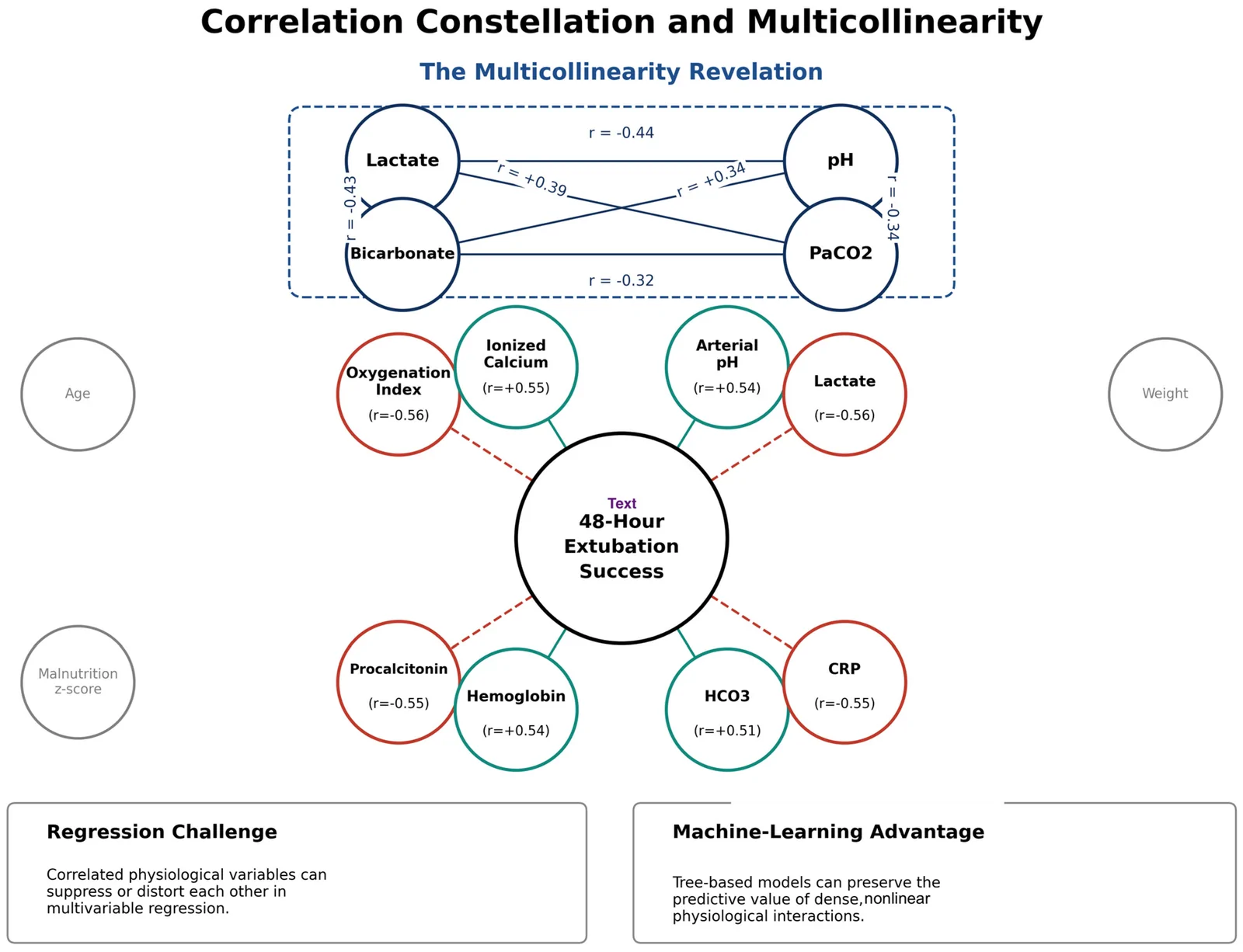
Correlation constellation and multicollinearity.

When extubation failure was treated as the positive class, the Random Forest achieved a PR-AUC of 0.866 (95% CI, 0.766–0.955), substantially exceeding the no-skill baseline of 0.126 corresponding to the observed 12.6% failure prevalence. K-means clustering of 12 standardized pre-extubation variables identified two phenotypes (silhouette coefficient: 0.657), with stable cluster assignments across resampling and alternative clustering analyses. A low-severity phenotype (n = 302, 88.6%) was characterized by a near-normal oxygenation index (5.9), lactate (1.5 mmol/L), and pH (7.39), with a 97% extubation success rate and no PICU mortality. A high-severity phenotype (n = 39, 11.4%) showed a markedly deranged oxygenation index (23.8), lactate (6.3 mmol/L), procalcitonin (30.2 ng/mL), CRP (217 mg/L), pH (7.17), and pSOFA (7.8), with only 10% extubation success and 100% PICU mortality (Figure 7). Mortality and post-extubation outcomes were not used for clustering; therefore, the observed mortality difference represents a post hoc association rather than an independent validation of the supervised model. Cross-tabulation of cluster membership against outcome events (Table 5) showed that 35 of 39 high-severity-cluster patients (89.7%) were reintubated within 48 h, compared with eight of 302 low-severity-cluster patients (2.6%); all 39 high-severity-cluster patients died during the PICU stay, compared with none of the 302 low-severity-cluster patients. This exact correspondence with mortality most likely reflects that the clustering variables are themselves established markers of physiological severity and organ dysfunction (Figure 8), rather than evidence that unsupervised clustering provides information independent of illness severity. Among the 39 patients in the high-severity cluster, SBT type was PSV + PEEP in 21 patients, CPAP in 16, and T-piece in two; median SBT duration was 90 min (range, 30–120), and the median number of SBTs was 2 (range, 1–3). In the low-severity cluster, SBT type was PSV + PEEP in 196 patients, CPAP in 88, and T-piece in 18. In the low-severity cluster, the median SBT duration was 90 min (range, 30–120), and the median number of SBTs was 2 (range, 1–3)

**Figure 9:**
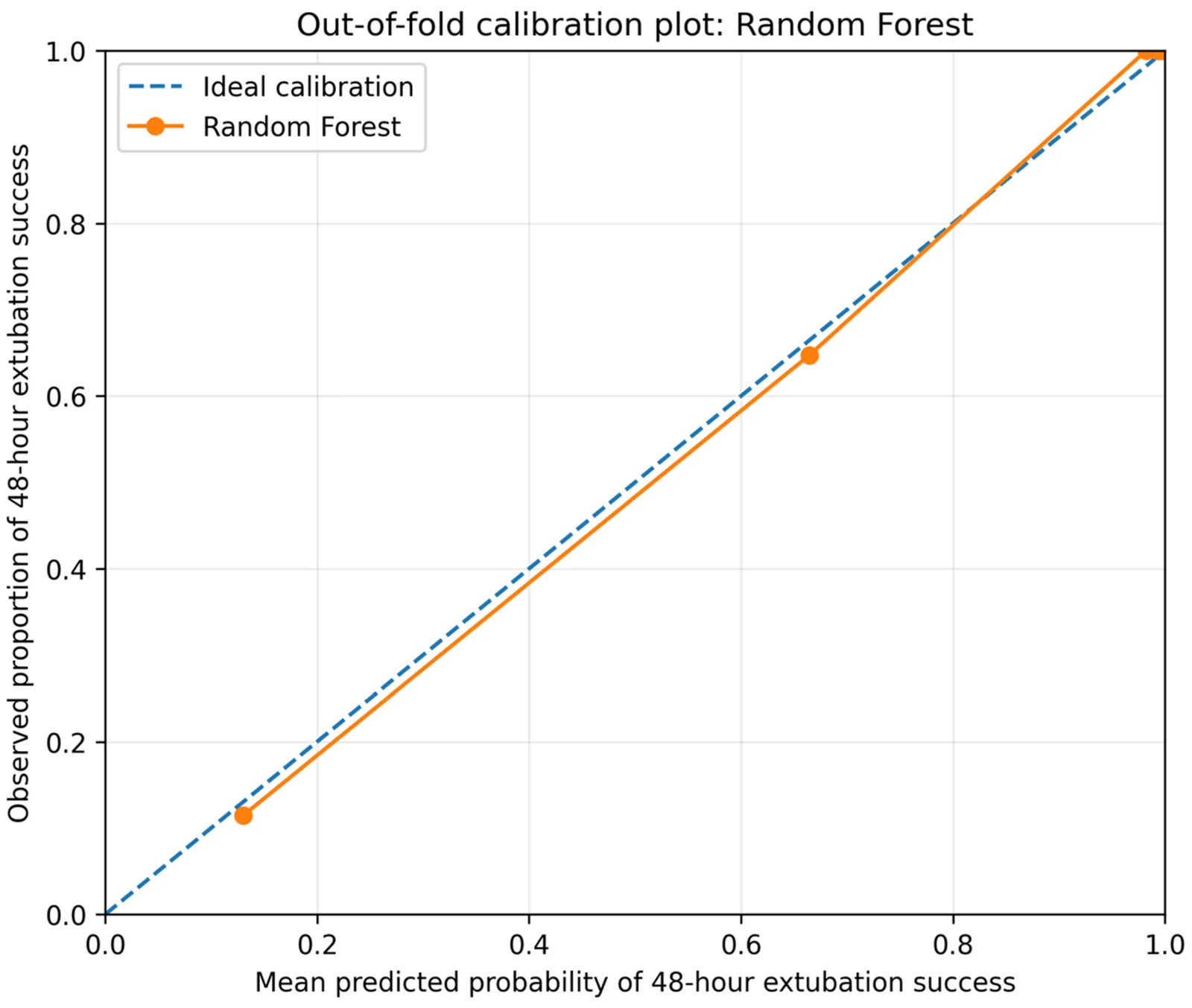
Out-of-fold calibration plot for the Random Forest model. Note: Observed 48 h extubation-success proportions are plotted against mean predicted probabilities obtained from stratified five-fold cross-validation; the dashed diagonal represents perfect calibration.

Calibration analysis of the pooled out-of-fold Random Forest predictions yielded a calibration-in-the-large of −0.010 and a calibration slope of 1.074. The Brier score was 0.032 (95% CI, 0.020–0.045), compared with 0.110 for the prevalence-only model, indicating improved probabilistic accuracy over the null benchmark (Figure 9).

Collectively, these findings support the hypothesis that a multidimensional artificial intelligence model integrating blood gas, metabolic, inflammatory, and ventilatory parameters can predict 48 h extubation success with high discriminative accuracy (Random Forest, AUC up to 0.983) in critically ill children. Arterial pH, oxygenation index, bicarbonate, ionized calcium, and procalcitonin were among the variables with the highest model-based importance scores, consistent with the physiological expectation that combined respiratory failure, tissue hypoperfusion, and systemic inflammation—rather than any isolated ventilator setting or demographic factor—were associated with extubation outcome in this cohort.

## 4. Discussion

**Figure 10:**
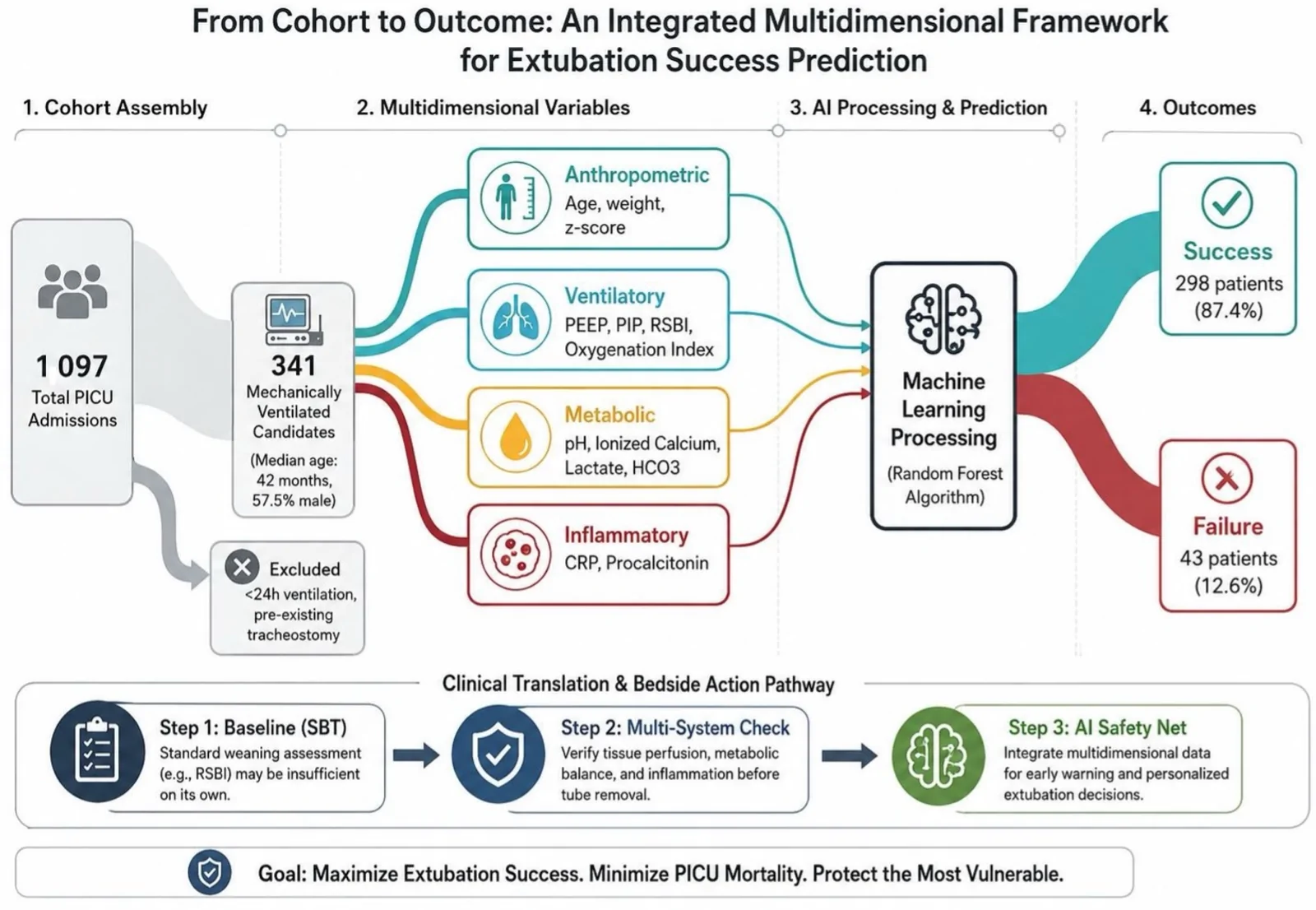
Integrated multidimensional framework for predicting 48-h extubation success.

In this single-center cohort of 341 mechanically ventilated children, 298 (87.4%) successfully extubated for at least 48 h, whereas 43 (12.6%) failed. The observed failure rate falls within the clinically important range reported in pediatric intensive care populations, although direct comparisons remain difficult because studies differ in age distribution, underlying diagnoses, duration of follow-up, post-extubation respiratory support, and whether death before reintubation is included in the endpoint. All aspects and main points supported in Figure 1 and Figure 10.

Ramnarayan et al. showed that post-extubation respiratory support strategy can influence subsequent liberation from respiratory support, while Burns et al. emphasized that successful spontaneous breathing testing does not, by itself, establish the ability to maintain airway patency, clear secretions, sustain respiratory muscle work, and preserve gas exchange after removal of the endotracheal tube [16,17].

Acute physiological status was a stronger predictor of extubation outcome than demographic or treatment-related variables, whereas primary diagnosis and chronic lung disease remained significant. This aligns with Ramnarayan et al., Burns et al., Emeriaud et al., and Hamill et al., who emphasized multidimensional neurological, airway, cardiorespiratory, and systemic assessment rather than isolated age-adjusted ventilator thresholds [16,17,18,19].

Oxygenation index had the second-highest Random Forest Gini importance score and is increasingly treated in the pediatric ARDS literature as a composite marker of oxygen-transfer failure and support intensity rather than as a single blood-gas value (18,19). Oxygenation index also ranked second in Random Forest importance, with an importance value of 0.090. Because this index incorporates mean airway pressure, FiO_2_, and PaO_2_, a high value represents both impaired oxygen transfer and the continued intensity of support required to maintain oxygenation. Contemporary pediatric acute respiratory distress guidance similarly treats the oxygenation index as a multidimensional measure of hypoxemic respiratory failure rather than an isolated blood gas measurement. Our finding that it outperformed the PaO2/FiO2 ratio alone (*p* = 0.020 vs. *p* < 0.001) is consistent with Emeriaud et al.’s argument that PALICC-2 criteria intentionally moved away from isolated oxygenation thresholds toward composite indices [16,18,19].

Ventilatory and acid–base abnormalities provided a second coherent physiological domain. Failure was associated with higher PaCO_2_ and lower arterial pH and bicarbonate; extubation success correlated negatively with PaCO_2_ (r =- 0.50) and positively with pH (r = 0.54) and bicarbonate (r = 0.51). Arterial pH had the highest Random Forest Gini importance score, with an importance of 0.118, while bicarbonate ranked third at 0.090 and PaCO_2_ ranked seventh at 0.071. These findings are physiologically credible because hypercapnia and acidemia can reflect inadequate alveolar ventilation, increased dead space ventilation, excessive respiratory workload, respiratory muscle fatigue, or unresolved pulmonary disease. Burns et al. and Trivedi et al. similarly concluded that respiratory indices must be interpreted as a combined physiological pattern rather than independent pass-or-fail criteria [17,20].

The RSBI was significantly higher in the failure group and negatively correlated with success (r =- 0.32); however, its Random Forest importance was only 0.030, ranking twelfth among the reported predictors. This comparatively modest contribution supports the position that the RSBI should supplement, rather than replace, multidomain clinical assessment. In the systematic review by Trivedi et al., which included 48 studies, the RSBI demonstrated a pooled sensitivity of approximately 83% but a specificity of only approximately 58%, indicating a limited ability to identify all patients who would fail liberation [5]. Jia et al. likewise reported substantial between-study heterogeneity and concluded that the RSBI alone is insufficiently reliable for extubation decision-making [21].

Extubation failure was associated with inflammation, impaired perfusion, and reduced metabolic reserve, reflected by higher lactate, procalcitonin, and CRP and lower albumin, hemoglobin, sodium, and ionized calcium. Consistent with Abbas et al., combined oxygen-delivery, perfusion, and organ-dysfunction markers were more informative than isolated measurements [22].

Neurological and global severity measurements further supported this interpretation. Extubation success correlated positively with the Glasgow Coma Scale score at r = 0.43 and sedation score at r = 0.40, while correlations were negative for pSOFA at r = −0.40 and PRISM-IV score at r = −0.27. These relationships are clinically plausible because sustained extubation requires respiratory drive, airway protection, effective cough, secretion handling, and coordinated breathing, none of which are captured completely by oxygenation or ventilator parameters. The absence of meaningful correlations with age, weight, and nutritional z-score therefore does not indicate that these characteristics are universally unimportant; rather, acute physiological derangement appeared to dominate prediction in this cohort. For Random Forest, the precision–recall AUC for the failure class was 0.866 (95% CI, 0.766–0.955), and the calibration slope and intercept (1.074 and −0.010, respectively) indicated good agreement between predicted and observed probabilities. Between-model differences were interpreted descriptively in view of the uncertainty surrounding the performance estimates. Inflammatory markers were also clinically relevant: extubation success correlated negatively with lactate (r = −0.56), procalcitonin (r = −0.55), and CRP (r = −0.55), consistent with Abbas et al., who reported that combined perfusion and organ-dysfunction measures were more informative than isolated parameters. However, as emphasized by Pappy et al. and van de Sande et al., strong internal performance requires external validation because correlated predictors, limited events, and model-selection optimism can inflate accuracy estimates [22,23,24]. The high internal discrimination should also be interpreted in the context of the marked physiological separation between outcome groups, including substantial differences in the oxygenation index, lactate, arterial pH, and inflammatory markers. This separation may have facilitated classification within the present cohort and should not be assumed to translate into comparable performance in external populations. Ghassemi et al. cautioned that model explanations and ranked feature importance can be misleading when interpreted as mechanisms, while DECIDE-AI recommends evaluating explanations within the intended clinical workflow rather than presenting them as causal evidence [25,26,27,28]. The discrepancy between Gini and held-out permutation rankings further indicates that feature-importance measures are model-dependent and should not be interpreted as evidence of independent clinical importance.

K-means clustering identified low- and high-severity respiratory–metabolic–inflammatory phenotypes (silhouette 0.657), with extubation success of 97% versus 10% and mortality of 0% versus 100%, respectively. This multidimensional separation is consistent with Saria et al. and Abbas et al., who showed that integrated physiological and oxygen-delivery patterns outperform isolated variables, and with Emeriaud et al. and Hamill et al., who emphasized the oxygenation index as a marker of pediatric respiratory severity. These phenotypes represent exploratory post hoc patterns rather than independent confirmation and require external validation [10,18,19,22,29,30,31]. Because the retrospective dataset did not preserve clinician-specific extubation indications or a standardized binary record of SBT success, the reasons for proceeding with planned extubation despite marked physiological derangement in individual high-severity patients could not be reconstructed reliably.

The main strengths of this study are the use of routinely available pre-extubation variables, the comparison of multiple supervised algorithms, the comprehensive assessment of discrimination and calibration, complementary feature-importance analyses, and exploratory unsupervised physiological phenotyping. The principal limitations are its retrospective single-center design, only 43 primary-outcome events, substantial class imbalance, possible variability in measurement timing, correlated predictors, potential model-selection optimism, the absence of external validation, and the possibility that management decisions influenced both predictor values and outcome [32,33,34,35]. The retrospective dataset did not permit reconstruction of exact measurement-to-extubation intervals for all predictors, potentially introducing variability in predictor timing.

Because model comparison and performance estimation were performed within the same cross-validation framework without a separate outer validation loop, the reported differences between algorithms may be optimistic. These results should therefore be interpreted as exploratory internal performance estimates that require confirmation through nested resampling and independent external validation. Accordingly, repeated cross-validation or resampling of the complete modeling pipeline would provide a more comprehensive assessment of model stability.

The retrospective dataset did not capture all extubation-readiness factors, including cough strength, secretion burden, upper airway or cuff-leak assessment, airway abnormalities, or the indication for individual extubation decisions. In addition, post-extubation NIV was recorded in 23.5% of patients, but the prophylactic versus rescue indication was unavailable; these unmeasured clinical factors may have influenced the risk of reintubation.

Future research should use a prospectively defined prediction time, locked pre-extubation variables, nested model tuning, temporal and geographic external validation, calibration updating, decision-curve analysis, subgroup fairness assessment, and silent prospective testing before clinical deployment. Reporting should follow TRIPOD + AI, early clinical evaluation should follow DECIDE-AI, and deployment planning should incorporate the FUTURE-AI principles of fairness, universality, traceability, usability, robustness, and explainability [12,36,37,38]. The present findings are therefore promising as an internal proof of concept, but the model should remain a research instrument until its calibration, utility, and safety are demonstrated across independent pediatric intensive care populations.

## 5. Conclusions

Extubation outcome in critically ill children appears to reflect an integrated respiratory, metabolic, inflammatory, and neurological state rather than any single conventional weaning index. Random Forest provided strong internally validated discrimination and identified clinically coherent model-based predictors, while exploratory k-means clustering identified distinct physiological phenotypes; however, these findings require prospective multicenter validation before bedside use.

## Figures and Tables

**Table 1:** Demographic, clinical, laboratory, and ventilator characteristics.

**Continuous Variables**			
**Variable**	**Mean ± SD**	**Median [IQR]**	**Min–Max**
Age (months)	67.57 ± 63.96	42 [12–119]	1–216
Weight (kg)	17.58 ± 12.31	13.4 [7.9–25]	2.9–61.6
Malnutrition (z-score)	−0.69 ± 1.8	−0.7 [−2.3–0.8]	−3.7–3.4
PICU length of stay (days)	13.4 ± 18.35	8 [3–15]	1–136
Hospital length of stay (days)	16.01 ± 18.5	10 [6–18]	1–141
Mechanical ventilation duration (days)	5.77 ± 7.41	3 [1–8]	1–53
Weaning duration (days)	1.74 ± 0.96	1 [1–2]	1–7
Sedation duration (days)	6.64 ± 7.57	4 [2–9]	1–55
Neuromuscular blockade (days)	1.53 ± 1.88	1 [0–2]	0–13
Vasoactive–Inotropic Score	4.1 ± 5.93	1.7 [0–5.9]	0–35.3
Heart rate (bpm)	115.27 ± 18.57	115 [100–130]	75–164
Respiratory rate (breaths/min)	27.14 ± 6.79	27 [22–33]	15–43
Glasgow Coma Scale	9.62 ± 2.32	10 [9–11]	3–15
Sedation score	15.3 ± 4.29	15 [12–18]	6–28
Hemoglobin (g/dL)	11.67 ± 1.55	11.9 [10.9–12.8]	7–13.8
C-reactive protein (mg/L)	45.41 ± 68.78	23.8 [11.9–35.9]	0–330.3
Procalcitonin (ng/mL)	4.18 ± 12.4	0.65 [0.36–0.93]	0.05–78.35
Albumin (g/dL)	3.91 ± 0.63	4 [3.7–4.4]	1.8–4.8
Lactate (mmol/L)	2.06 ± 1.74	1.5 [1.1–1.9]	0.8–9
Arterial pH	7.37 ± 0.08	7.39 [7.36–7.42]	7.05–7.45
PaCO_2_ (mmHg)	42.43 ± 6.52	40.9 [38.4–44]	35.1–69.7
HCO_3_^−^ (mmol/L)	23.98 ± 3.07	24.5 [22.9–26.2]	14.1–28
Sodium (mmol/L)	139.45 ± 3.78	139.7 [137.3–142.5]	128.1–145
Potassium (mmol/L)	4.2 ± 0.46	4.2 [3.8–4.5]	3.1–5.8
Ionized calcium (mmol/L)	1.16 ± 0.09	1.17 [1.12–1.23]	0.9–1.3
Magnesium (mg/dL)	1.95 ± 0.21	1.96 [1.78–2.12]	1.33–2.3
Phosphorus (mg/dL)	4.29 ± 1.08	4.3 [3.6–5.1]	1.5–7.6
PaO_2_ (mmHg)	96.31 ± 27.02	94.6 [77.9–112]	13.8–196.1
FiO_2_ (fraction)	0.28 ± 0.07	0.3 [0.21–0.35]	0.21–0.4
PaO_2_/FiO_2_ ratio	351.26 ± 108.57	337.71 [267.14–419.52]	39.43–729.05
SaO_2_ (%)	94.7 ± 1.57	94.5 [93.5–95.8]	92–100
PEEP (cmH_2_O)	5.72 ± 1.19	5 [5–6]	4–12
PIP (cmH_2_O)	20.76 ± 4.09	20.8 [18.1–23.4]	12–31.2
Peak PIP (cmH_2_O)	28.59 ± 11.86	30.5 [23.9–36.5]	2–58.6
Tidal volume (mL/kg)	7.07 ± 0.98	7.1 [6.4–7.7]	4.2–10
Rapid Shallow Breathing Index	6.35 ± 1.86	6.3 [5.2–7.3]	2–14
Oxygenation Index	7.96 ± 6.34	6.2 [4.2–7.9]	2.3–31.8
SBT duration (min)	81.47 ± 34.03	90 [60–120]	30–120
Number of SBTs	1.93 ± 0.82	2 [1–3]	1–3
PRISM-IV score	6.01 ± 5.25	5 [3–8]	0–44
PRISM-IV predicted mortality (%)	6.01 ± 13.38	1.55 [0.65–5]	0.1–93.65
pSOFA score	3.65 ± 2.69	3 [2–5]	0–14
**Categorical Variables and Clinical Outcomes**			
**Variable**	**Category**	**n**	**%**
Sex	Female	145	42.5
	Male	196	57.5
Primary diagnosis	Healthy/No comorbidity	84	24.6
	Respiratory	100	29.3
	Neurological	89	26.1
	Cardiac	3	0.9
	Infection/Sepsis	65	19.1
Chronic lung disease	No	300	88
	Yes	41	12
Vasoactive drug use	No	225	66
	Yes	116	34
Sepsis	No	252	73.9
	Yes	89	26.1
Acute kidney injury	No	299	87.7
	Yes	42	12.3
CRRT	No	326	95.6
	Yes	15	4.4
Weaning method	Gradual pressure reduction (invasive)	168	49.3
	CPAP	152	44.6
	T-piece	21	6.2
SBT type	PSV + PEEP	217	63.6
	CPAP	104	30.5
	T-piece	20	5.9
Extubation success (48 h)	No	43	12.6
	Yes	298	87.4
Reintubation during overall follow-up, n (%)	No	277	81.2
	Yes Reintubation within 48 h: 43 (12.6%) Reintubation after 48 h: 21 (6.2%)	64	18.8
NIV after extubation	No	261	76.5
	Yes	80	23.5
Post-extubation stridor	No	295	86.5
	Yes	46	13.5
Tracheostomy (outcome)	No	301	88.5
	Yes	39	11.5
PICU mortality	No	302	88.6
	Yes	39	11.4

**Table 2:** Bivariate comparison by 48-h extubation outcome.

Continuous Variables (Mann–Whitney U Test, Non-Parametric Due to Non-Normal Distribution on Shapiro–Wilk Testing):				
Variable	Success (n = 298) Median [IQR]	Failure (n = 43) Median [IQR]	U Statistic	*p*-Value
Oxygenation Index *	5.70 [4.00–7.10]	22.20 [17.90–26.30]	216	<0.001
Lactate (mmol/L) *	1.40 [1.10–1.80]	5.20 [3.45–8.05]	231	<0.001
Procalcitonin (ng/mL) *	0.55 [0.33–0.81]	13.16 [6.29–39.99]	317.5	<0.001
C-reactive protein (mg/L) *	20.60 [10.45–30.75]	191.30 [107.80–261.15]	327.5	<0.001
Ionized calcium (mmol/L) *	1.18 [1.14–1.24]	1.00 [0.93–1.06]	12,475.5	<0.001
Arterial pH *	7.39 [7.37–7.42]	7.18 [7.12–7.29]	12,459	<0.001
Hemoglobin (g/dL) *	12.10 [11.30–13.00]	8.90 [7.60–9.85]	12,400	<0.001
Albumin (g/dL) *	4.10 [3.80–4.40]	2.70 [2.35–3.05]	12,238	<0.001
HCO_3_ (mmol/L) *	24.80 [23.40–26.40]	17.40 [16.30–19.95]	12,090	<0.001
PaCO_2_ (mmHg) *	40.15 [38.02–42.90]	53.80 [47.70–62.20]	822.5	<0.001
Sodium (mmol/L) *	140.25 [138.30–142.80]	133.80 [130.35–135.25]	11,969.5	<0.001
Glasgow Coma Scale *	10.00 [9.00–11.00]	6.00 [4.50–9.00]	11,111	<0.001
Sedation score *	16.00 [13.00–19.00]	10.00 [9.00–12.00]	10,858.5	<0.001
pSOFA score *	3.00 [1.90–4.00]	7.00 [5.00–9.00]	1991.5	<0.001
Phosphorus (mg/dL) *	4.50 [3.80–5.20]	2.80 [2.25–3.50]	10,764	<0.001
Magnesium (mg/dL) *	1.99 [1.83–2.13]	1.73 [1.60–1.88]	10,642	<0.001
PEEP (cmH_2_O) *	5.00 [5.00–6.00]	7.00 [6.00–8.00]	2831	<0.001
Rapid Shallow Breathing Index *	6.10 [5.10–6.97]	8.50 [7.20–9.80]	2893	<0.001
PRISM-IV predicted mortality (%) *	1.39 [0.58–3.68]	10.00 [1.50–33.33]	3173	<0.001
Heart rate (bpm) *	113.50 [98.00–127.00]	133.00 [112.00–143.00]	3298	<0.001
PRISM-IV score *	5.00 [3.00–7.00]	10.00 [4.50–17.00]	3394	<0.001
Respiratory rate (breaths/min) *	26.00 [21.00–32.00]	31.00 [24.50–35.50]	4493.5	0.002
SBT duration (min) *	90.00 [60.00–120.00]	90.00 [75.00–120.00]	4909	0.010
Potassium (mmol/L) *	4.10 [3.80–4.50]	4.50 [4.00–5.00]	4882.5	0.011
PaO_2_/FiO_2_ ratio *	342.43 [271.00–423.81]	316.75 [245.12–381.86]	7816.5	0.020
FiO_2_ (fraction) *	0.30 [0.21–0.35]	0.35 [0.21–0.35]	5112.5	0.025
Mechanical ventilation duration (days)	3.00 [1.00–7.00]	5.00 [1.00–11.00]	5261.5	0.051
Sedation duration (days)	4.00 [2.00–8.00]	6.00 [2.00–12.50]	5293	0.064
Neuromuscular blockade (days)	1.00 [0.00–2.00]	1.00 [1.00–2.00]	5521	0.127
Hospital length of stay (days)	10.00 [6.00–18.00]	12.00 [7.00–20.00]	5543	0.152
PICU length of stay (days)	7.00 [3.00–15.00]	11.00 [4.50–17.00]	5586.5	0.174
Vasoactive–Inotropic Score	1.60 [0.00–5.80]	2.50 [0.00–7.65]	5684.5	0.214
Malnutrition (z-score)	−0.60 [−2.20–0.70]	−2.20 [−2.90–1.20]	7028.5	0.304
Peak PIP (cmH_2_O)	30.00 [22.25–37.00]	31.00 [29.00–34.00]	5870.5	0.375
Number of SBTs	2.00 [1.00–3.00]	2.00 [1.00–3.00]	6871.5	0.415
PIP (cmH_2_O)	20.70 [18.02–23.40]	21.00 [18.70–23.90]	6031.5	0.535
PaO_2_ (mmHg)	95.40 [77.93–111.97]	91.50 [73.05–110.75]	6758.5	0.561
Weight (kg)	13.45 [7.90–25.23]	12.00 [6.75–22.70]	6679	0.653
Weaning duration (days)	1.00 [1.00–2.00]	1.00 [1.00–3.00]	6255.5	0.784
Age (months)	44.00 [12.00–122.75]	36.00 [12.00–105.50]	6289.5	0.846
SaO_2_ (%)	94.50 [93.60–95.80]	94.30 [93.50–95.85]	6480	0.904
Tidal volume (mL/kg)	7.05 [6.43–7.70]	7.20 [6.40–7.70]	6475.5	0.910
Categorical variables (chi-square or Fisher exact test where expected cell counts < 5):				
Variable			Test used	*p*-value
Primary diagnosis *			chi2 (low-cell)	<0.001
Chronic lung disease *			chi-square	0.03
Sepsis				0.224
Sex				0.289
SBT type			chi2 (low-cell)	0.384
Weaning method				0.457
Vasoactive drug use			chi-square	0.519
Acute kidney injury				0.919
CRRT			Fisher exact	1.000

* p < 0.05, statistically significant.

**Table 3:** Prediction of 48-h extubation success (5-fold stratified cross-validation).

Model	AUC (95% CI)	Accuracy (95% CI)	Sensitivity (95% CI)	Specificity (95% CI)	PPV (95% CI)	F1 (95% CI)	Brier (95% CI)
Logistic Regression (L2)	0.924 (0.866–0.971)	0.938 (0.912–0.962)	0.966 (0.946–0.987)	0.744 (0.605–0.860)	0.963 (0.944–0.980)	0.965 (0.949–0.978)	0.050 (0.031–0.070)
Random Forest	0.983 (0.971–0.993)	0.959 (0.938–0.977)	0.980 (0.963–0.993)	0.814 (0.698–0.930)	0.973 (0.957–0.990)	0.977 (0.965–0.987)	0.032 (0.020–0.045)
Gradient Boosting	0.975 (0.955–0.990)	0.950 (0.927–0.971)	0.966 (0.946–0.987)	0.837 (0.721–0.930)	0.976 (0.959–0.990)	0.971 (0.958–0.983)	0.041 (0.024–0.061)
Support Vector Machine (RBF)	0.953 (0.914–0.983)	0.965 (0.944–0.982)	0.987 (0.973–0.997)	0.814 (0.698–0.930)	0.974 (0.957–0.990)	0.980 (0.968–0.990)	0.034 (0.019–0.051)
Fold-level ROC-AUC values across stratified five-fold cross-validation							
Model	Fold 1	Fold 2	Fold 3	Fold 4	Fold 5	Reported pooled AUC	
Logistic Regression	0.935	0.902	0.963	1.000	0.844	0.924	
Random Forest	0.989	0.987	0.977	1.000	0.966	0.983	
Gradient Boosting	0.993	0.983	0.975	1.000	0.966	0.975	
Support Vector Machine	0.939	0.923	0.981	1.000	0.927	0.953	

Values are point estimates with 95% percentile confidence intervals derived from 3000 stratified bootstrap resamples of pooled out-of-fold predictions. The observed success/failure class distribution was preserved during bootstrap resampling. Classification metrics were calculated using a probability threshold of 0.50; extubation success was treated as the positive class.

**Table 4:** Random Forest feature importance.

Top Predictors: Random Forest Importance Ranking		
Predictor	RF Importance (True Rank)	
Ionized calcium (mmol/L)	0.088 (#4)	
Arterial pH	0.118 (#1)	
Hemoglobin (g/dL)	0.036 (#11)	
Sodium (mmol/L)	0.044 (#10)	
Albumin (g/dL)	0.058 (#9)	
Rapid Shallow Breathing Index	0.030 (#12)	
Procalcitonin (ng/mL)	0.087 (#5)	
HCO_3_ (mmol/L)	0.090 (#3)	
Oxygenation Index	0.090 (#2)	
Lactate (mmol/L)	0.074 (#6)	
C-reactive protein (mg/L)	0.069 (#8)	
PaCO_2_ (mmHg)	0.071 (#7)	
Unsupervised K-Means Clustering—Physiologic Severity Phenotypes (scikit-learn)		
Cluster characterization (mean values) *:		
Variable	Cluster 0: Low-Severity	Cluster 1: High-Severity
Number of patients, n	302	39
Oxygenation Index, mean	5.92	23.81
Lactate, mean	1.52	6.25
Procalcitonin, mean	0.83	30.16
CRP, mean	23.22	217.22
pH, mean	7.39	7.17
PaCO_2_, mean	40.59	56.64
HCO_3_^−^, mean	24.87	17.07
pSOFA score, mean	3.11	7.84
PRISM-IV score, mean	4.97	14.08
GCS score, mean	10.12	5.77
RSBI, mean	6.03	8.83
PEEP, mean	5.51	7.38
Mechanical ventilation duration, mean days	5.43	8.46
Extubation success rate	97.00%	10.00%
PICU mortality rate	0.00%	100.00%
Silhouette scores by k (k = 2 selected—highest score) **		
k	Silhouette score	
2	0.657	
3	0.152	
4	0.129	
5	0.119	

Outcome predicted: Extubation_Success (48 h). Features: all baseline/intra-ICU clinical, laboratory, and ventilator parameters.* Clustering variables (standardized): oxygenation index, lactate, procalcitonin, CRP, pH, PaCO_2_. ** Interpretation: Cluster 0 (n = 302) shows near-normal physiology with 97% extubation success and 0% mortality. Cluster 1 (n = 39) shows combined respiratory/metabolic/inflammatory derangement with only 10% success and 100% mortality—the marked outcome differences between clusters represent post hoc associations and should not be interpreted as independent validation of the supervised models

**Table 5:** Model interpretability and cluster validation: comparison of feature-importance methods and outcome cross-tabulation.

* **Comparison of Random Forest Gini Importance and Permutation Importance**				
**Predictor**	**Gini Rank**	**Gini Importance**	**Permutation Rank (Held out)**	**Permutation Importance (95% CI)**
RSBI	12	0.030	1	0.0040 (0.0009–0.0072)
Arterial pH	1	0.118	2	0.0026 (−0.0026 to 0.0078)
Lactate	6	0.074	3	0.0016 (0.0000–0.0031)
Ionized Calcium	4	0.088	4	0.0015 (0.0006–0.0024)
PaCO_2_	7	0.071	5	0.0011 (−0.0007 to 0.0029)
Oxygenation Index	2	0.090	6	0.0007 (−0.00003 to 0.0014)
CRP	8	0.069	7	0.0006 (−0.0004 to 0.0015)
Procalcitonin	5	0.087	8	0.0005 (−0.0001 to 0.0011)
Bicarbonate (HCO_3_)	3	0.090	15	0.0001 (−0.0004 to 0.0007)
** **Cross-tabulation of unsupervised k-means cluster membership against 48 h reintubation, later reintubation, and PICU mortality**				
**Outcome**		**Cluster 0—Low severity (n = 302)**	**Cluster 1—High severity (n = 39)**	**Total (n = 341)**
Reintubated ≤48 h (primary failure)		8 (2.6%)	35 (89.7%)	43 (12.6%)
Reintubated >48 h (secondary event)		19 (6.3%)	2 (5.1%)	21 (6.2%)
PICU mortality		0 (0.0%)	39 (100.0%)	39 (11.4%)
Died without reintubation		0 (0.0%)	2 (5.1%)	2 (0.6%)

* Permutation importance = mean decrease in held-out AUC (5-fold cross-validation, 30 repeats per fold, held-out AUC decrease). CIs crossing zero indicate no distinguishable individual effect. ** Clusters from k-means (k = 2) on 12 pre-extubation variables. Percentages within cluster. Mortality/reintubation not used in clustering.

## Data Availability

The datasets used and/or analyzed during the current study are available from the corresponding author on reasonable request.
